# Identification of novel *FOXP1* variants in four unrelated patients with intellectual disability and speech impairment

**DOI:** 10.3389/fneur.2026.1743089

**Published:** 2026-02-04

**Authors:** Jingjing Xiang, Jun Mao, Qin Zhang, Qingxia Meng

**Affiliations:** The Affiliated Suzhou Hospital of Nanjing Medical University, Suzhou Municipal Hospital, Gusu School, Nanjing Medical University, Suzhou, Jiangsu, China

**Keywords:** FOXP1 syndrome, whole exome sequencing, intellectual disability, speech impairment, variants, RNA sequencing

## Abstract

**Background:**

To document the clinical phenotypes and identify the genetic causes of four unrelated children with intellectual disability and speech impairment.

**Methods:**

Trio-based whole exome sequencing (Trios) was performed for four probands and their parents. Identified variants underwent pathogenicity assessment utilizing *in silico* protein structure prediction and RNA analysis.

**Results:**

Trios revealed four novel *de novo* heterozygous *FOXP1* variants: a frameshift variant c.1909dup (p.Glu637Glyfs^*^10), two missense variants c.1568T>C (p.Phe523Ser) and c.1541G>T (p.Arg514Leu), and a splicing variant c.1653-34_1653-25delTTTAACTTTG, all confirmed by Sanger sequencing. Protein structure analysis predicted that the missense variants, located within the forkhead box (FOX) domain, are likely to impair protein function. RNA analysis demonstrated that the splicing variant c.1653-34_1653-25delTTTAACTTTG induced skipping of exons 18 and 19, resulting in an in-frame deletion of 64 amino acids (p.Asn511_Gln574del).

**Conclusion:**

Our findings expand the mutational spectrum of *FOXP1* and underscore the utility of *in silico* protein structure prediction and RNA analysis in the classification of variants of uncertain significance.

## 1 Introduction

*FOXP1* (*Forkhead Box P1*) gene, located on chromosomal region 3p14.1, encodes a transcriptional repressor. As a member of the forkhead box (FOX) family of transcriptional factors, the FOXP1 protein is expressed widely in human tissues and plays important roles in the development of brain, lung, heart and immune system (1–5).

*De novo* heterozygous mutations or deletions of *FOXP1* are associated with FOXP1 Syndrome (OMIM 613670), a neurodevelopmental disorder characterized by global developmental delay, intellectual disability, language impairment, behavior abnormalities and facial dysmorphisms (6, 7). The first description of FOXP1 syndrome was a child with a *de novo* 785 kb deletion of the 3p14.1p13 region encompassing the entire *FOXP1* gene and adjacent genomic regions (8). Then Hamdan et al. (9) reported two unrelated cases with intragenic *FOXP1* mutations: one carrying a multi-exon deletion (exons 4–14) and another with a nonsense variant. By 2023, over 200 individuals have been identified with FOXP1 syndrome (10). However, FOXP1 syndrome exhibits substantial phenotypic variability, and no clear genotype-phenotype correlations have been established (6, 7, 11).

Whole exome sequencing (WES) has been recommended as a first-tier diagnostic test for neurodevelopmental disorders, especially when presentations are atypical or non-specific (12). In this study, we characterize the clinical phenotypes of four pediatric patients harboring novel *FOXP1* variants identified via WES. The variants comprise one frameshift, two missense, and one splicing variant, none of which have been reported previously. To assess their pathogenicity, we performed *in silico* protein structure prediction and RNA analysis. This integrated approach establishes a functional validation framework for variants of uncertain significance.

## 2 Materials and methods

### 2.1 Patients

This study enrolled four pediatric patients presenting unexplained developmental delay and intellectual disability referred to our center for reproduction and genetics, the affiliated Suzhou hospital of Nanjing medical university, Suzhou, Jiangsu, China. There were no siblings for any of the four probands in this study. Table 1 summarizes the clinical characteristics of four patients, including age, gender, and phenotypic features documented by pediatric specialists and clinical geneticists. Approval for this study was obtained by the ethics committee of the Affiliated Suzhou Hospital of Nanjing Medical University. Written informed consent was obtained from all participants' legal guardians prior to study inclusion.

**Table 1:** Phenotypic description of four patients with *FOXP1* variants.

	**Patient 1**	**Patient 2**	**Patient 3**	**Patient 4**
Age	5 years 6 months	6 years 10 months	6 years 2 months	7 years 6 months
Sex	Male	Male	Female	Female
Broad, prominent forehead	+	+	+	+
Downslanting palpebral fissures	+	–	–	–
Hypertelorism	+	+	–	–
Strabismus	–	–	+	–
Flat nasal bridge	+	+	+	–
Single transverse palmar crease	–	–	+	–
Hypertonia	–	–	–	+
Abnormal brain imaging	+	–	+	+
Abnormal electroencephalography	–	–	+	–
Behavior abnormalities	–	+	–	–
Global developmental delay	+	+	+	+
Motor delay	+	+	+	+
Intellectual disability	+	+	+	+
Speech and language delay	+	+	+	+

### 2.2 Whole exome sequencing and data analysis

Genomic DNA was extracted from the whole blood samples of four patients and their parents respectively. Whole exome capture was performed by xGen^®^ Exome Research Panel v2.0 (Integrated DNA Technologies, USA) and sequenced using DNBSEQ-T7 platform (MGI Tech, China). Sequencing reads were aligned to the Ensemble human reference genome (hg19/GRCh37) using Burrows-Wheeler Aligner. Single-nucleotide variants (SNVs) and small insertions or deletions (Indels) were identified by GATK (https://software.broadinstitute.org/gatk/). Population allele frequencies of the variants were obtained by searching in the dbSNP (http://www.ncbi.nlm.nih.gov/SNP/), 1000 Genomes Project database (http://www.1000genomes.org/) and the Genome Aggregation Database (gnomAD) (http://gnomad.broadinstitute.org/). Missense variants were assessed for pathogenicity using REVEL (https://sites.google.com/site/revelgenomics/), and the REVEL score for a missense variant can range from 0 to 1, with higher scores reflecting greater likelihood of disease-causing. Potential splicing effect of splicing variants was predicted by SpliceAI (https://spliceailookup.broadinstitute.org/), and the score of SpliceAI can range from 0 to 1, which can be interpreted as the probability of the variant being splice-altering. Clinical relevance was evaluated by searching against public databases such as OMIM (http://www.omim.org), ClinVar (http://www.ncbi.nlm.nih.gov/clinvar), and PubMed (http://www.ncbi.nlm.nih.gov/pubmed). The candidate variants were classified according to the Standards and Guidelines for the Interpretation of Sequence Variants released by the American College of Medical Genetics and Genomics and the Association for Molecular Pathology (13).

### 2.3 Sanger sequencing

To validate the WES findings, targeted polymerase chain reaction (PCR) amplification of identified candidate variants was performed using genomic DNA from the patients and their parents respectively. Purified PCR products underwent bidirectional Sanger sequencing by an ABI 3500 Genetic Analyzer (Applied Biosystems, USA). Sequence chromatograms were analyzed and compared against corresponding GenBank reference sequences of *FOXP1* gene (NM_001349338.3) to verify mutation positions.

### 2.4 Multiple sequence alignment and structural prediction

To assess amino acid conservation, homologous protein sequences of FOXP1 were downloaded from the NCBI database and aligned using ClustalW. The human FOXP1 protein structure (Q9H334) was obtained from UniProt (https://www.uniprot.org/). The structure was visualized in ChimeraX using secondary structure coloring, and annotated with IBS 2.0. Comparative structural analysis was then conducted between the wild-type and mutant proteins.

### 2.5 RNA analysis

Total RNA was extracted from whole blood by the RNAprep Pure Hi-Blood Kit (Tiangen, China). For RNA sequencing (RNA-seq), cDNA library construction was performed using the mRNA-seq Lib Prep Kit for Illumina (ABclonal, China). DNA nanoballs were prepared using the DNBSEQ-T7RS High-throughput Sequencing Set (FCL PE150) V2.0 (MGI Tech, China), and sequenced on the DNBSEQ-T7 platform (MGI Tech, China). After removing adapter sequences and low-quality reads, the sequencing reads were aligned to the reference genome GRCh37/hg19 using HISAT2. Subsequent variant analysis was performed using GATK to call SNPs and InDels. Alternative splicing events were analyzed using SplAdder. Fusion gene detection was carried out using STAR-Fusion.

To confirm the results of RNA-seq, total RNA was reverse-transcribed into complementary DNA (cDNA) using HiScript III 1st Strand cDNA Synthesis Kit (+gDNA wiper) (Vazyme, China). The fragments spanning the variants were PCR-amplified from cDNA templates, analyzed by agarose gel electrophoresis followed by Sanger sequencing. For TA cloning, purified PCR products were ligated into pTG19-T vector and transformed into DH5α competent cells. Plasmid DNA was isolated from 3 to 5 positive clones and verified by Sanger sequencing.

## 3 Results

### 3.1 Clinical description of patients

**Figure 1:**
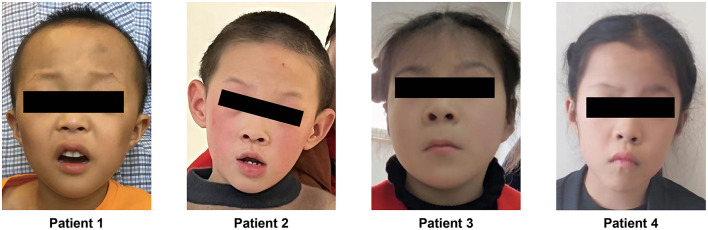
Facial characteristics of four unrelated patients with novel *FOXP1* variants.

Patient 1 is a 5-year-old boy born to healthy nonconsanguineous Chinese parents (Figure 1, Table 1). After birth, the child gradually exhibited delayed responses and global developmental delay. Brain MRI at age 7 months revealed increased cortical convolutions in the cerebral hemispheres, suggestive of polymicrogyria, and a subsequent MRI at age 2 years showed a thin corpus callosum. At the age of 3, he was able to walk but demonstrated persistently poor language development. He was diagnosed with cerebral palsy at this time. He also exhibited mild facial dysmorphic features such as broad forehead, thin, long eyebrows, downslanting palpebral fissures, epicanthus palpebralis, hypertelorism and a low nasal bridge. Genetic investigations, including karyotyping and chromosomal microarray analysis yielded normal results.

Patient 2 is a 6-year-old boy born to healthy non-consanguineous Chinese parents (Figure 1, Table 1). The child was delivered via cesarean section with a birth weight of 3,100 g. There was no history of feeding difficulties or sleep disturbances. Developmental delay was first noted around 5 months of age. He was unable to lift his head at 5 months, could not sit independently at approximately 11 months, and achieved independent walking at 2.5 years. Language development was limited to babbling “da-da” at 10 months with no significant progress thereafter, and he currently lacks purposeful verbal expression. Fine motor skills are delayed, and currently he is unable to hold a spoon correctly. A developmental assessment at 3.5 years yielded a Developmental Quotient (DQ) of 28 (Gross Motor: 41, Fine Motor: 27, Adaptive: 26, Language: 14, Social: 20). He exhibits oral stereotypies (persistent vocalizations, repetitive tongue play) and self-injurious head-hitting when agitated. Dysmorphic features include long face, broad forehead, highly-arched and sparse eyebrows, narrow palpebral fissures, hypertelorism, a flat nasal bridge and large protruding ears. Investigations including tandem mass spectrometry and urinary gas chromatography/mass spectrometry (GC/MS) around 1 year of age, and a brain MRI at 3 years showed no significant abnormalities.

Patient 3 is a 6-year-old girl born to healthy non-consanguineous Chinese parents (Figure 1, Table 1). She was delivered full-term by cesarean section, with low birth weight (2.4 kg). The neonatal period was unremarkable. Developmental delays became gradually apparent from 9 months of age. The infant was unable to roll over at 9 months and brain MRI revealed a small arachnoid cyst in the cisterna magna. She could walk by age 3. At the age of 5, her Intelligence Quotient (IQ) was 51. Electroencephalography (EEG) showed abnormalities: paroxysmal bursts of high-amplitude (3–4 Hz) sharp-and-slow wave complexes and spike-and-slow wave complexes were observed over bilateral frontal poles, frontal regions, and midline frontal areas during all sleep stages. Tandem mass spectrometry and urinary GC/MS were normal. Blood ammonia levels, thyroid function tests, and lactate levels were also within normal ranges. Physical examination noted broad forehead, strabismus, a flat nasal bridge, long philtrum, thin lips, large ears, and single transverse palmar crease.

Patient 4 is a 7-year-old girl born to healthy nonconsanguineous Chinese parents (Figure 1, Table 1). At 6 months of age, abnormal hand postures and poor grasping ability were noted, accompanied by increased muscle tone. She was able to walk at 17 months. Developmental assessments at 5 years revealed a DQ of 52 and an IQ of 49. Brain MRI demonstrated widening of the frontoparietal extracerebral space. Currently, the child exhibits global developmental delay, intellectual disability, and hypertonia. Characteristic facial features include long face, broad forehead, highly-arched and sparse eyebrows, epicanthus palpebralis, and a long nose.

### 3.2 Genetic analysis

**Figure 2:**
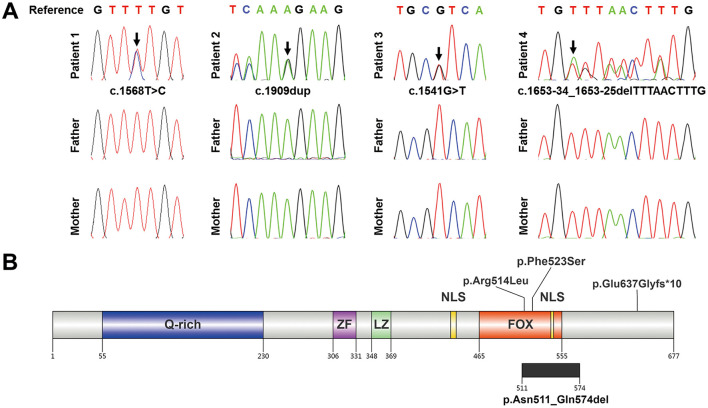
Identification of *de novo FOXP1* variants in four patients. **(A)** Confirmation of *de novo FOXP1* variants. Sanger sequencing confirmed the presence of heterozygous *FOXP1* variants in four patients, as indicated by black arrows respectively. These variants were absent in parental samples, confirming their *de novo* origin. **(B)** Schematic diagram of the human FOXP1 protein. Major domains are labeled, including an N-terminal glutamine-rich region (Q-rich), a zinc finger (ZF) domain, a leucine zipper (LZ) domain, and a C-terminal forkhead box (FOX) domain. Two nuclear localization signals (NLS) are also indicated. Variants described in this study are shown above and below the schematic, as indicated.

Whole exome sequencing was conducted on family trios to identify candidate variants. These variants were subsequently evaluated based on clinical relevance, pathogenicity evidence, ACMG category, and inheritance mode of associated disease. Ultimately, we identified previously unreported *de novo* heterozygous *FOXP1* variants in all four patients, which were confirmed by Sanger sequencing respectively (Figure 2A). Patient 1 carries a missense variant c.1568T>C in exon 18 of the *FOXP1* gene, causing a substitution from phenylalanine to serine of FOXP1 protein (p.Phe523Ser). For patient 2, a novel heterozygous variant c.1909dup in exon 21 of the *FOXP1* gene was identified, leading to a frameshift and a premature stop codon (p.Glu637Glyfs^*^10). Patient 3 also carries a missense variant c.1541G>T in exon 18 of the *FOXP1* gene, leading to a substitution from arginine to leucine of FOXP1 protein (p.Arg514Leu) (Figure 2B). Patient 4 carries a splicing variant c.1653-34_1653-25delTTTAACTTTG in intron 18 of the *FOXP1* gene (Figure 2A).

These four variants were not recorded in the dbSNP, 1000 Genomes Project database, or gnomAD (Accessed: 25 December 2025). The c.1909dup variant, located in the last exon of the *FOXP1* gene, is not predicted to undergo nonsense mediated decay (NMD). This prediction is supported by RNA-seq data showing that the variant was expressed in 40.2% (99/246) of total *FOXP1* transcripts in Patient 2 (Supplementary Figure 1). This frameshift introduces a premature stop codon after nine novel amino acids, resulting in a truncated protein that lacks < 10% of the wild-type FOXP1 protein sequence. Furthermore, patient 2′s phenotype is highly suggestive of a monogenic disorder and closely matches the characteristic features of FOXP1 syndrome. According to the ACMG variant classification guideline (13), the c.1909dup variant could be classified as likely pathogenic with 2 moderate (PS2_Moderate and PVS1_Moderate) and 2 supporting (PM2_Supporting and PP4) evidence.

**Figure 3:**
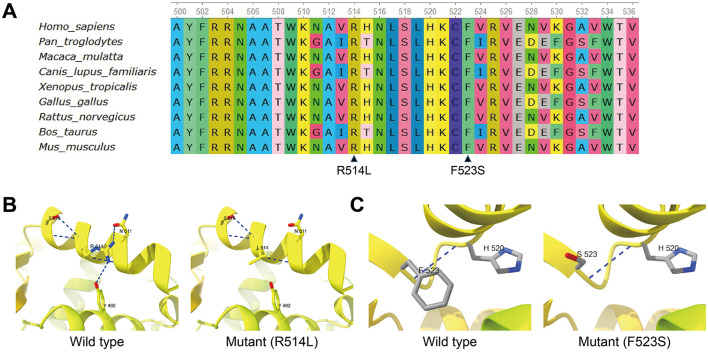
Missense mutations in the FOX domain. **(A)** Multiple sequence alignment of human FOXP1 protein and its orthologs in different species showing the conserved positions of residues R514 and F523. **(B)** Predicted structures of wild-type FOXP1 and the R514L mutant protein. In the wild-type protein, R514 is predicted to form hydrogen bonds with N511 and Y492. These interactions are disrupted by the R514L substitution in the R514L mutant protein. **(C)** Predicted structures of wild-type FOXP1 and the F523S mutant protein. In the F523S mutant protein, the F523S mutation leads to the loss of the aromatic ring present in phenylalanine.

Quantitative analysis by RNA-seq revealed that the c.1541G>T variant accounted for 50.5% (210/416) of all *FOXP1* transcripts in Patient 3 (Supplementary Figure 1). The missense variant c.1541G>T (p.Arg514Leu) is predicted to be deleterious by REVEL with a score of 0.961, and the missense variant c.1568T>C (p.Phe523Ser) is predicted to be deleterious by REVEL with a score of 0.969. Furthermore, sequence alignment of human FOXP1 protein and its othologs in different species demonstrated that the residues Arg514 and Phe523 are highly conserved among species (Figure 3A). Protein structure analysis suggested that the Arg514Leu substitution in FOXP1 may impair hydrogen bonding, potentially perturbing protein structure and function (Figure 3B). Although the Phe523Ser substitution is not predicted to reduce hydrogen bonding, replacing the hydrophobic phenylalanine with a hydrophilic serine and eliminating its aromatic ring could significantly alter local hydrophobicity and steric interactions, thereby affecting structure and function (Figure 3C). In addition, two different missense variants c.1541G>A (p.Arg514His) and c.1540C>T (p.Arg514Cys) occurring at the same position Arg514 were determined to be pathogenic by previous studies (14, 15). According to the ACMG variant classification guideline (13), the c.1568T>C variant could be classified as uncertain significance with 1 moderate (PS2_Moderate) and 2 supporting (PM2_Supporting and PP3) evidence, and the c.1541G>T variant could be classified as likely pathogenic with 2 moderate (PS2_Moderate and PM5) and 2 supporting (PM2_Supporting and PP3) evidence.

**Figure 4:**
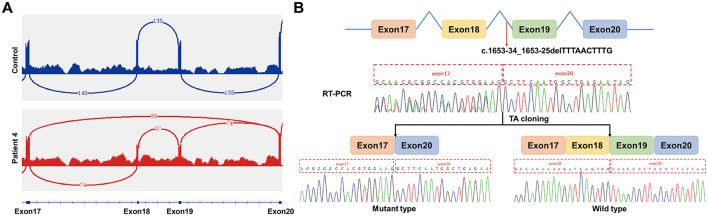
RNA analysis of the splicing variant in patient 4. **(A)** RNA-seq data of peripheral blood demonstrated in-frame skipping of exons 18 and 19 in some transcripts caused by the c.1653-34_1653-25delTTTAACTTTG variant of *FOXP1* in patient 4, compared to normal splicing seen in a control case. **(B)** Schematic diagram of *FOXP1*. Exons are represented by boxes and introns by lines. The splicing variant c.1653-34_1653-25delTTTAACTTTG is indicated by a red arrow. Sanger sequencing of RT-PCR products revealed overlapping chromatograms upstream of exon 20, suggesting mixed transcripts. TA-cloning and sequencing of individual clones subsequently identified two transcript species: the wild type and an aberrant transcript lacking exons 18 and 19.

SpliceAI predicts that the splicing variant c.1653-34_1653-25delTTTAACTTTG disrupts the acceptor splice site (score = 0.43), potentially leading to abnormal RNA splicing. To evaluate the effect of this splicing variant, RNA processing was analyzed in patient 4 using RNA extracted from peripheral blood. RNA-seq showed that the c.1653-34_1653-25delTTTAACTTTG variant led to skipping of exons 18 and 19 in approximately 1/2 of all *FOXP1* transcripts in patient 4 (Figure 4A). Further RT-PCR analysisand direct Sanger sequencing of the RT-PCR products showed overlapping sequence chromatograms before exon 20, suggesting heterogeneous transcripts. Subsequent TA-cloning and sequencing of individual clones confirmed two transcript species: a wild-type transcript and an aberrant transcript resulting from the skipping of exons 18 and 19 (Figure 4B). Skipping of exons 18 and 19 is predicted to cause an in-frame deletion (p.Asn511_Gln574del) in the FOXP1 protein without disrupting the open reading frame. The deleted region, encompassing a portion of the FOX DNA-binding domain and the nuclear localization signal 2 (NLS2), constitutes < 10% of the wild-type protein sequence. However, both domains are critical for FOXP1 function (Figure 2B). According to the ACMG variant classification guideline (13), the c.1653-34_1653-25delTTTAACTTTG variant could be classified as likely pathogenic with 1 strong (PVS1_Strong), 1 moderate (PS2_Moderate) and 1 supporting (PM2_Supporting) evidence.

## 4 Discussion

FOXP1 belongs to the FOXP subfamily within the FOX transcription factor family. This subfamily comprises four members (FOXP1-FOXP4), each linked to distinct disorders. Heterozygous pathogenic variants in *FOXP1* are associated with FOXP1 syndrome (OMIM 613670), manifesting global developmental delay, intellectual disability and language impairment. Pathogenic variants in *FOXP2* cause speech-language disorder-1 (OMIM 602081), characterized by severe orofacial dyspraxia. *FOXP3* mutations result in IPEX syndrome (OMIM 304790), an X-linked disorder featuring immune dysregulation, polyendocrinopathy, and enteropathy. Heterozygous variants of *FOXP4* has been reported to cause a developmental disorder with speech/language delays and multiple congenital abnormalities (16).

The expression of FOXP1, FOXP2, and FOXP4 display distinct cell type-specific spatiotemporal patterns in the brain (17). This heterogeneity in expression likely underpins the diverse clinical phenotypes observed across different *FOXP* mutations. For instance, pathogenic variants in *FOXP1* are associated with global impairments in neural development and function, whereas mutations in *FOXP2* primarily result in specific deficits in speech and language (18). Moreover, these FOXP proteins are co-expressed in the striatum, a critical region for human speech and language (17). Of note, speech impairment is a prominent feature in disorders associated with *FOXP1, FOXP2*, and *FOXP4*. Studies in zebra finches have shown that FoxP1, FoxP2, and FoxP4 are coexpressed, can form homodimers and heterodimers with each other. And knockdown of FoxP1, FoxP2, or FoxP4 in the striatal song nucleus Area X of zebra finches, resulted in impaired vocal learning (19).

To date, 186 pathogenic or likely pathogenic short variants of *FOXP1* (< 50 bps), including small InDels, frameshift, missense, nonsense, and splice site variants, have been recorded in ClinVar (Accessed: 25 December 2025). In this study, we identified four novel heterozygous *FOXP1* variants in four unrelated patients: a frameshift variant, two missense variants, and a splicing variant. None of the four patients exhibited colic or abnormal vital reflexes during the neonatal period, and the phenotypes associated with FOXP1 syndrome emerged during early childhood. The phenotypic characteristics shared by all four patients in this study, including global developmental delay, motor delay, intellectual disability, speech/language delay and prominent forehead, (Figure 1, Table 1), which are consistent with previous reports of FOXP1 Syndrome (20, 21). However, phenotypic variability exists beyond cognitive and speech-related manifestations. Brain MRI revealed abnormalities in Patients 1, 3, and 4, but Patient 2′s brian MRI scan was normal. A flat nasal bridge was present Patients 1, 2, and 3, and hypertelorism was observed in patients 1 and 2. Moreover, each of the following features was unique to a single patient: downslanting palpebral fissures (Patient 1), behavior abnormalities (Patient 2), strabismus, a single transverse palmar crease, abnormal electroencephalography (Patient 3), and hypertonia (Patient 4) (Table 1). This variability is consistent with the broad phenotypic spectrum reported in FOXP1-related disorders, where wide phenotypic variation has been noted even among four patients with the same mutation c.1573C>T (p.Arg525^*^) of *FOXP1* (11). While previous studies reported no significant differences in clinical presentation between patient groups with FOXP1 syndrome harboring missense mutation vs. premature truncating mutations in large cohorts (7, 11), our small sample size does not allow for such an analysis.

The identification of gross deletions encompassing *FOXP1* and loss-of-function variants suggests that *FOXP1* haploinsufficiency is the likely pathogenic mechanism underlying FOXP1 syndrome. The frameshift variant c.1909dup (p.Glu637Glyfs^*^10) detected in patient 2 is shown to escape NMD, as demonstraed by RNA-seq data (Supplementary Figure 1). The resulting truncated protein retains all functional domains, suggesting that this variant may impair protein stability or exert a dominant-negative effect by binding wild-type FOXP1 and FOXP2 proteins.

Two missense variants identified in this study, c.1541G>T (p.Arg514Leu) and c.1568T>C (p.Phe523Ser), are located within the FOX domain of FOXP1 (Figure 2B). This localization is consistent with a previous report indicating that 11 out of 13 missense variants cluster within the FOX domain (7), which is crucial for DNA recognition, binding, and protein-protein interactions. Both Arg514 and Phe523 residues are highly conserved across species (Figure 3A). Sollis et al. reported recurrent missense variants at the same Arg514 residue: c.1541G>A (p.Arg514His) in three unrelated patients and c.1540C>T (p.Arg514Cys) in one patient with FOXP1 syndrome (14, 15). Given these findings along with our identification of the c.1541G>T (p.Arg514Leu) variant, the Arg514 site emerges as a strong candidate for a mutation hotspot within FOXP1. The pathogenic FOXP1 variants p.Arg514His and p.Arg514Cys have been reported to disrupt subcellular localization, form cytoplasmic and nuclear aggregates, and severely abolish transcriptional repression (14, 15). Protein structure analysis predicts that the p.Arg514Leu variant reduces hydrogen bonding, suggesting comparable functional impairments. The missense variant c.1568T>C (p.Phe523Ser) has been recorded in ClinVar (Accessed: 25 December 2025), where it has conflicting interpretations of pathogenicity. One submission classified it as “Likely pathogenic”, whereas two submissions classified it as “Uncertain significance”. The Phe523Ser substitution, while not predicted to reduce hydrogen bonding, replaces a hydrophobic phenylalanine with a hydrophilic serine and eliminates its aromatic ring. This alteration may disrupt local hydrophobicity and introduce steric clashes, potentially compromising structural integrity and functional capacity. Supporting this, two pathogenic variants of the conserved phenylalanine residues at the equivalent positions of other FOX proteins have been reported, including p.Phe106Leu in FOXF1 (NP_001442.2) of a patient with alveolar capillary dysplasia with misalignment of pulmonary veins and p.Phe129Leu in FOXE3 (NP_036318.1) of a patient with congenital bilateral, total sclerocornea, aphakia, and microphthalmia (22, 23). Moreover, Johnson et al. have reported three patients with a missense c.1574G>A (p.Arg525Gln) mutation of *FOXP1*, which resulted in diminished transcriptional repression but normal nuclear localization (24).

The c.1653-34_1653-25delTTTAACTTTG variant identified in Patient 4 lies beyond canonical splice sites, proximal to the intron 18 acceptor site. RNA studies by RNA-seq and RT-PCR confirmed its pathogenicity, revealing concurrent skipping of exons 18 and 19. This results in an in-frame deletion (p.Asn511_Gln574del) within the FOXP1 protein. SpliceAI predicts disruption of the intron 18 acceptor site by this variant; however, our experimental data indicated additional disruption of the intron 18 donor splice site, which likely leads to exon 18 skipping. The FOX domain is evolutionarily conserved, including five α-helices (H1-H5) and three β sheets (β1–β3), where H3, β2 and β3 mediate DNA recognition and binding, H2 and H4 enable dimer formation (25). The deleted region (p.Asn511_Gln574del) encompasses critical functional domains: the entire H5 helix, β2–β3 sheets, and part of H3 helix, along with NLS2, which may cause impaired DNA interaction and aberrant nuclear localization.

## 5 Conclusion

In conclusion, four novel *de novo* heterozygous *FOXP1* variants were identified via WES in four unrelated children with neurodevelopmental delay and speech impairment, thereby expanding the known *FOXP1* mutation spectrum. This study further emphasizes the value of employing protein structure prediction and RNA analysis for the assessment of variant pathogenicity.

## Data Availability

All datasets generated for this study are included in the article. The genotyping data for this article are not publicly available to assure patient confidentiality and participant privacy. Requests to access the datasets should be directed to zhangq110004@163.com.
